# Positional multi-length and mutual-attention network for epileptic seizure classification

**DOI:** 10.3389/fncom.2024.1358780

**Published:** 2024-01-25

**Authors:** Guokai Zhang, Aiming Zhang, Huan Liu, Jihao Luo, Jianqing Chen

**Affiliations:** ^1^School of Optical-Electrical and Computer Engineering, University of Shanghai for Science and Technology, Shanghai, China; ^2^Department of Hematology, Affiliated Qingdao Central Hospital of Qingdao University, Qingdao Cancer Hospital, Qingdao, China; ^3^School of Computing, National University of Singapore, Singapore, Singapore; ^4^Department of Otolaryngology, Head and Neck Surgery, Shanghai Ninth People's Hospital, Affiliated to Shanghai Jiaotong University School of Medicine, Shanghai, China

**Keywords:** EEG signal, feature encoding, multi-length, feature reinforcement, deep learning

## Abstract

The automatic classification of epilepsy electroencephalogram (EEG) signals plays a crucial role in diagnosing neurological diseases. Although promising results have been achieved by deep learning methods in this task, capturing the minute abnormal characteristics, contextual information, and long dependencies of EEG signals remains a challenge. To address this challenge, a positional multi-length and mutual-attention (PMM) network is proposed for the automatic classification of epilepsy EEG signals. The PMM network incorporates a positional feature encoding process that extracts minute abnormal characteristics from the EEG signal and utilizes a multi-length feature learning process with a hierarchy residual dilated LSTM (RDLSTM) to capture long contextual dependencies. Furthermore, a mutual-attention feature reinforcement process is employed to learn the global and relative feature dependencies and enhance the discriminative abilities of the network. To validate the effectiveness PMM network, we conduct extensive experiments on the public dataset and the experimental results demonstrate the superior performance of the PMM network compared to state-of-the-art methods.

## 1 Introduction

Epilepsy is a prevalent neurological disease worldwide, affecting individuals' cognitive abilities and presenting risks of sudden falls or fatality (Rajinikanth et al., 2022). To mitigate epilepsy risks, the analysis of Electroencephalography (EEG) signals is the most effective approach to identify real-time neural disorder activity. However, EEG events often exhibit subtle amplitude variations, and manual detection of EEG signals is time-consuming, prone to errors, and requires specialized expertise. Thus, the significance of automatic EEG diagnosis lies in its capacity to analyze EEG signals with efficiency and accuracy, facilitating the timely detection of epilepsy (Xin et al., 2022; Wu et al., 2023).

Early studies on automatic EEG diagnosis focused on using hand-engineered low-level features, such as spectral, temporal, low-frequency, and high-frequency features, to achieve automatic classification of EEG signals (Liu et al., 2022). For instance, Lemm et al. (2005) applied spatio-spectral filters to mitigate the impact of noisy, non-stationary, and contaminated information in EEG signals, thereby improving classification performance. Meng et al. (2014) proposed a novel approach that learned spatial and spectral features and optimized the loss function by calculating the mutual information between the learned spectral features and class labels. Additionally, to assess the impact of different frequency sub-bands on EEG classification accuracy (Tsipouras, 2019), multiple sub-bands were combined as feature vectors. In another work, Qi et al. (2015) introduced regularized spatio-temporal filtering to classify EEG signals using supervised optimization algorithms. Jrad (2016) utilized high-frequency oscillations to extract relevant features, which were then input into a support vector machine for classifying different EEG events. Furthermore, Gao et al. (2019) developed a multi-scale information analysis model that utilized high-frequency EEG oscillations to recognize emotional states. Despite the success of these approaches, the subjective selection of hand-engineered features typically requires domain knowledge and may not capture the full range of characteristics present in input EEG signals.

Recently, with the great success of the convolutional neural network (CNN) on a broad array of medical image analysis, a large body of work in this area has been considered (Chen et al., 2021, 2023). Compared with the traditional hand-engineered methods, the CNN-based ones have the advantage of extracting more complicated and discriminative characteristics from the medical image. For instance, Zheng and Lu (2015) adopted the deep belief networks (DBNs) as the main detection architecture to train differential features for the automatic detection of the seizure. Regarding temporal features, Kasabov and Capecci (2015) designed a spiking neural network architecture that extracted spatio-temporal features for detecting and interpreting EEG signals. In Liu M. et al. (2020), it used the pre-trained CNN models to extract deep features and then adopted the cartesian K-means algorithm to conduct the semi-supervised learning on the EEG data. Furthermore, the unsupervised learning method (Chai et al., 2016) which combined the auto-encoder network with a subspace alignment solution into a unified framework was developed for analyzing the EEG data. Some other works, such as Qiu et al. (2018) and Liu J. et al. (2020) utilized the sparse autoencoder with different classifiers to jointly detect the seizure signal. Moreover, to improve the performance of the classification model, Yuan et al. (2018a) proposed a multi-view CNN model which aimed to learn the brain seizure from input multi-channel signals. Similarly, in Yuan et al. (2018b), it further developed a novel channel-aware attention network for multi-channel EEG seizure detection by using CNNs. Hossain et al. (2019) proposed a model to extract the spectral, temporal features and then input them to the classifier for EEG seizure classification. For learning the multi-scale features from the EEG, Zhang et al. (2020) designed a multi-scale non-local (MNL) network with two special layers to achieve promising classification results of the seizure. Additionally, some other works (Aliyu and Lim, 2021; Hussain et al., 2021; Saichand, 2021) adopted the long short-term memory (LSTM) to overcome the vanishing gradient problem of the recurrent neural network and boost the feature extraction ability of the EEG signal data.

Despite the promising results shown by CNN-based methods for EEG signal classification, three major challenges still need to be addressed. Firstly, seizures in EEG signals often exhibit subtle abnormal characteristics that can pose challenges for feature extraction, potentially impacting the performance of classification models. Secondly, the extraction of long contextual dependencies is crucial for effective EEG signal classification, but the use of LSTM for this purpose is impeded by limited receptive fields, which compromises their ability to capture necessary contextual information. Lastly, it is worth noting that previous works have paid relatively less attention to the incorporation of global relative dependencies in EEG signal analysis, which could offer valuable discriminative information crucial for improving classification accuracy. To tackle these challenges, we propose a novel approach called the positional multi-length and mutual-attention (PMM) network. The PMM network comprises three main processes: positional feature encoding, multi-length feature learning, and mutual-attention feature reinforcement. In the positional feature encoding process, minute abnormal characteristics from the shallow layers of the network are captured through the utilization of residual positional attention. This facilitates the PMM network in focusing on and extracting crucial information associated with those characteristics. The multi-length feature learning process employs a stacking of hierarchical residual dilated (RD) LSTMs to acquire long contextual dependencies within the EEG signal. By doing so, the network becomes adept at capturing temporal patterns across various time scales and effectively modeling the relationships between distant time steps. To further fortify the features, a mutual-attention feature reinforcement process is introduced. This process delves into both the global discriminative and relative dependencies present in the EEG signal. It selectively enhances informative features while simultaneously suppressing irrelevant ones, thereby enhancing the overall discriminative power of the network. Incorporating these three processes into the PMM network enables it to capture minute abnormal characteristics, long contextual dependencies, and global discriminative and relative dependencies simultaneously, resulting in a significant improvement in classifying EEG signals. Overall, the main contributions of this paper can be summarized as follows:

(1) A novel PMM network is proposed for the automatic classification of epilepsy seizures from EEG signals, with the incorporation of positional feature encoding to improve the extraction of minute abnormal characteristics from the EEG signals.

(2) In the proposed multi-length feature learning process, hierarchical RDLSTMs are used to capture long contextual dependencies from the EEG signal. Additionally, mutual-attention feature reinforcement is employed to jointly explore global discriminative features and relative dependencies simultaneously.

(3) Extensive experiments are conducted on the publicly available dataset. The results of the comparative analysis demonstrate that competitive performance is achieved by our proposed PMM network when compared to other state-of-the-art methods.

The remainder of the paper is organized as follows. Section 2 provides an introduction to the main method used in our proposed network. In Section 3, we provide a detailed description of the experimental data utilized in our study. Section 4 covers the implementation details, evaluation metrics, and a series of experiments conducted to evaluate the performance of our proposed approach. Finally, in Section 6, we summarize the findings of our study and provide a conclusion.

## 2 Method

**Figure 1:**
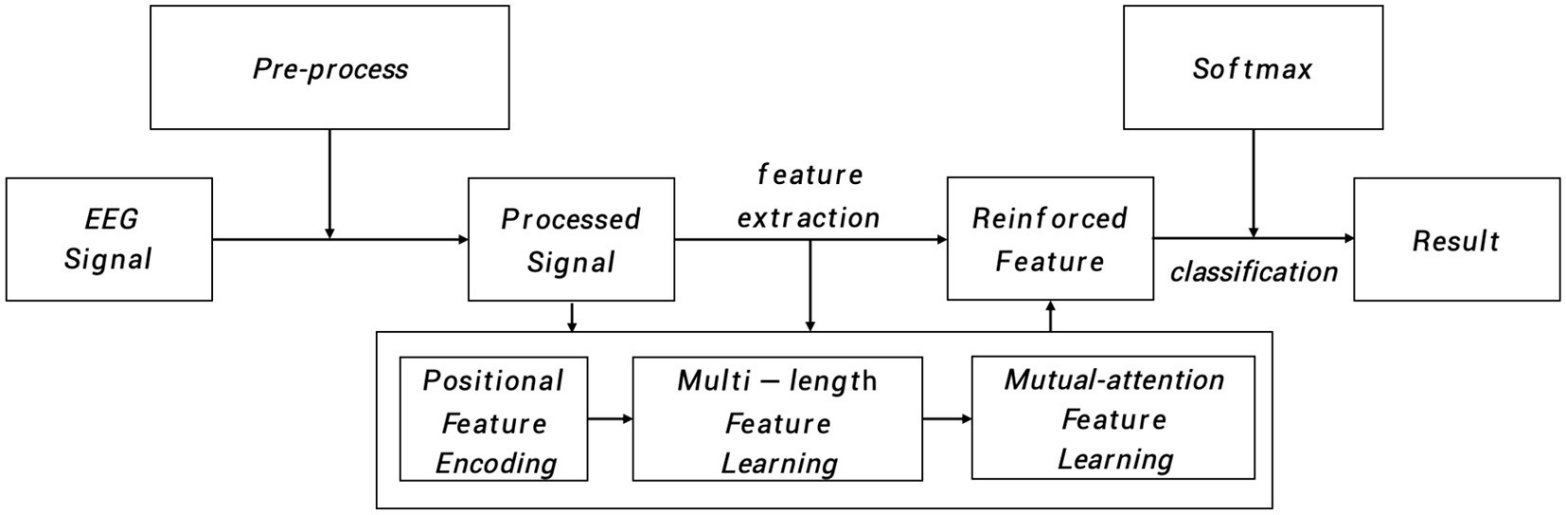
The PMM network systematically analyzes EEG signals through pre-process. The processed signal then undergoes positional feature encoding, multi-length feature learning, and mutual-attention feature reinforcement. Finally, the reinforced features are processed in a dense layer with softmax activation to generate the predicted result.

**Figure 2:**
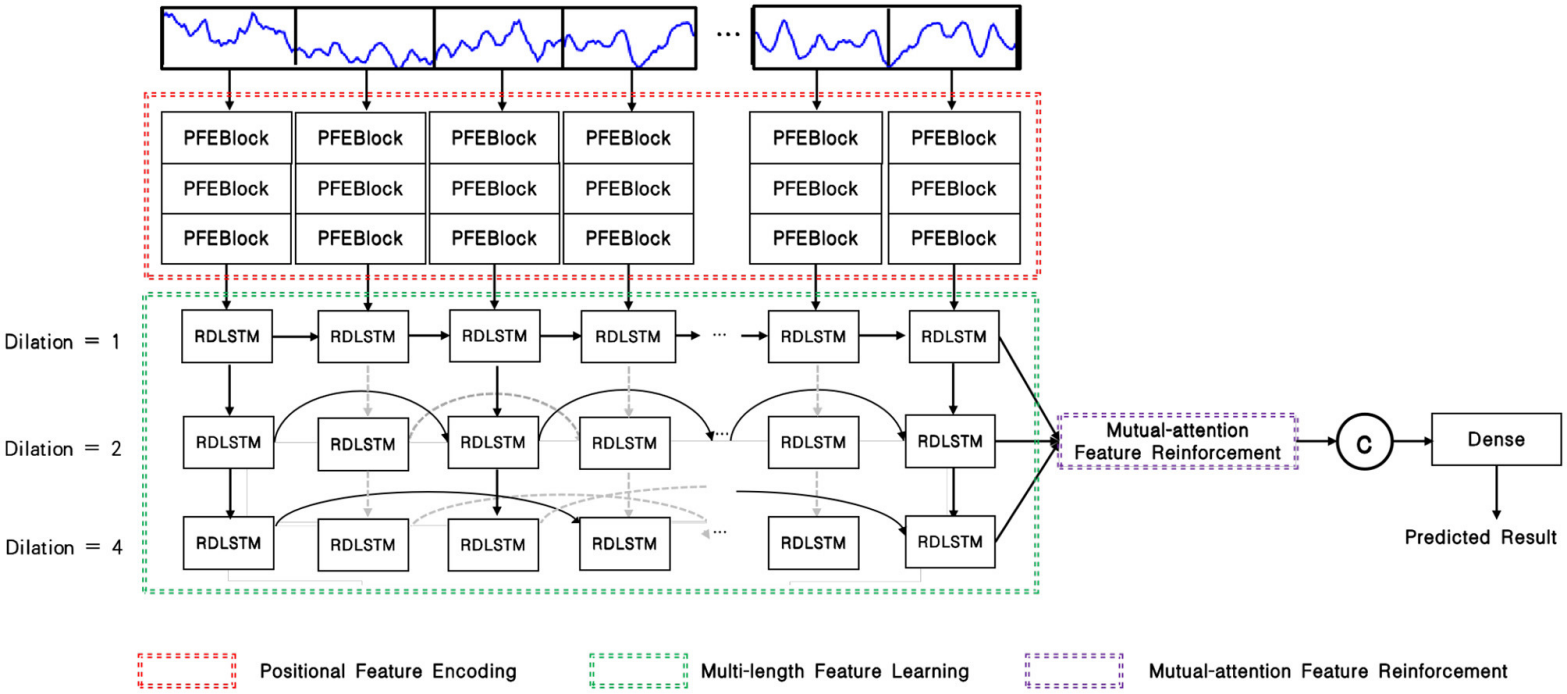
Overview of the PMM network, the PFEBlocks capture minute abnormal features, while the stacked RDLSTMs capture long contextual dependencies from the processed EEG signal. Additionally, the mutual-attention feature reinforcement further enhances the network's capability by extracting global discriminative features and relative dependencies.

As depicted in Figure 1, the input EEG signal undergoes an initial pre-processing step to obtain the processed signal. This processed signal is then fed into the positional feature encoding module, which extracts subtle abnormal characteristics from the shallow layers. Subsequently, multi-length feature learning and mutual-attention feature reinforcement are employed to enhance the classification of the PMM network. Finally, the reinforced feature is delivered to the dense layer with the softmax activation function to generate the predicted result. To provide a more specific overview, the main architecture of the proposed PMM network is illustrated in Figure 2. Initially, the input processed signal undergoes a positional feature encoding process, employing multiple positional feature encoding blocks (PFEBlocks) to capture minute abnormal features. Next, a multi-length feature learning process utilizing stacked RDLSTMs is employed to capture long contextual dependencies from the EEG signal. Furthermore, the network includes a mutual-attention feature reinforcement mechanism, which enables the extraction of both global discriminative features and relative dependencies, enhancing the network's overall capability in these aspects. In the following subsections, we will provide more detailed descriptions of positional feature encoding, multi-length feature learning, and mutual-attention feature reinforcement.

### 2.1 Positional feature encoding

**Figure 3:**
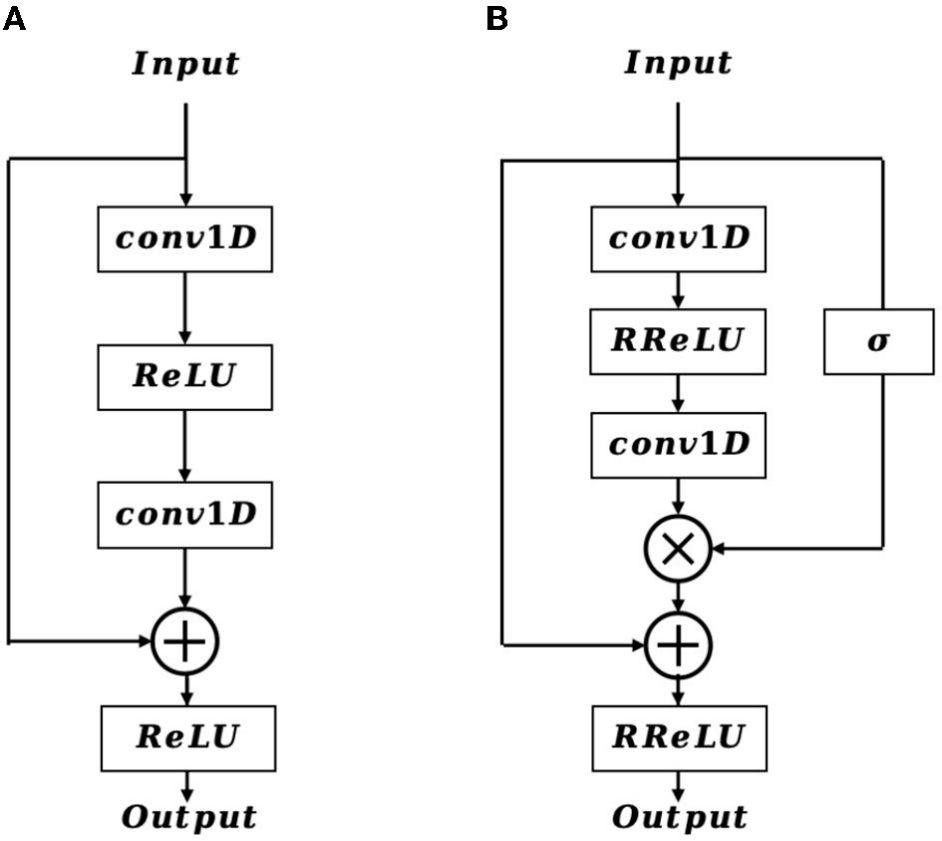
The structure of residual block **(A)** and positional feature encoding block **(B)**.

In the shallow layers of the network, the extracted feature map contains crucial details of the EEG signal that are vital for accurate EEG classification. Inspired by the structure of the residual block (Figure 3A), we incorporate a positional feature encoding block (Figure 3B) during feature encoding to automatically extract informative detail representations from the EEG signal. Considering the input of the positional feature encoding as **F_e_**, it first passes through the 1D convolution layer, which can be defined as:


(1) $$\begin{array}{l} {\text{X}}_{\text{o}}=\underset{\text{R}}{\sum }\text{W}*{\text{F}}_{e}+\text{b} \end{array}$$

where **X_o_** is the output feature vector, **R** denotes the receptive field, **W** and **b** is the weighted parameter and bias, respectively. After that, a randomized leaky rectified linear unit (RReLU) nonlinear activation function is employed, which is formulated as:


(2) $$\begin{array}{l} \text{y}=\{\begin{array}{cc} \text{x} & \text{if }\text{x}\geq 0\\ \text{a}\text{x} & \text{if }\text{x}<0 \end{array} \end{array}$$

where **x** is the input value, **a** represents a random number gained from the uniform distribution **U(p, q)**, and it is given as:


(3) $$\begin{array}{l} \text{a}\sim \text{U}\left(\text{p},\text{q}\right),\text{p}<\text{q}\text{ and }\text{p},\text{q}\in \left[0,1\right) \end{array}$$

Next, an extra 1D convolution layer is adopted to further refine the output feature from the RReLU activation function, resulting in ${\text{X}}_{\text{o}}^{\prime }$. In contrast to the traditional residual block, the proposed positional feature encoding block applies a sigmoid operation on **F_e_** to obtain the position weight matrix **W_pos_**. The formulation of **W_pos_** is defined as follows:


(4) $$\begin{array}{l} {\text{W}}_{\text{pos}}=\frac{e^{{\text{F}}_{\text{e}}}}{e^{{\text{F}}_{\text{e}}}+1} \end{array}$$

Afterward, we multiply **W_pos_** with ${\text{X}}_{\text{o}}^{\prime }$, and then add it to **F_e_** by a residual connection, therefore the final output feature map of **F_o_** is formulated as:


(5) $$\begin{array}{l} {\text{F}}_{\text{o}}={\text{W}}_{\text{pos}}*{\text{X}}_{\text{o}}^{\prime }+{\text{F}}_{\text{e}} \end{array}$$

Subsequently, the positional feature encoding block is followed by a 1D max-pooling layer, which downsamples the resolution of **F_o_**, and the resulting feature map is then further refined and enhanced through multi-length feature learning.

### 2.2 Multi-length feature learning

**Figure 4:**
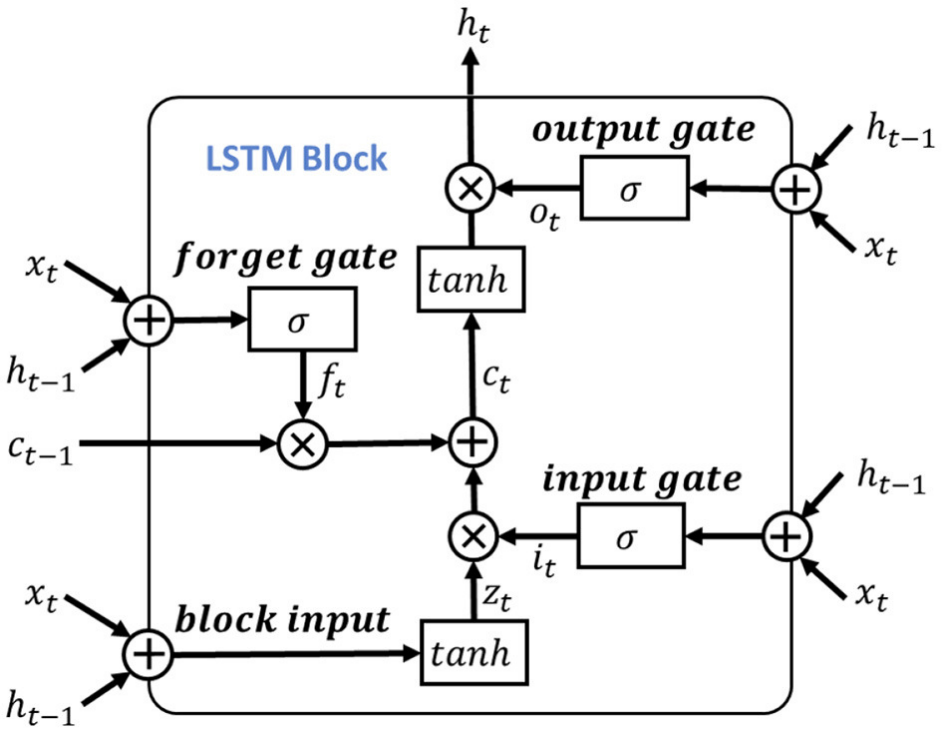
The structure of LSTM block.

**Figure 5:**
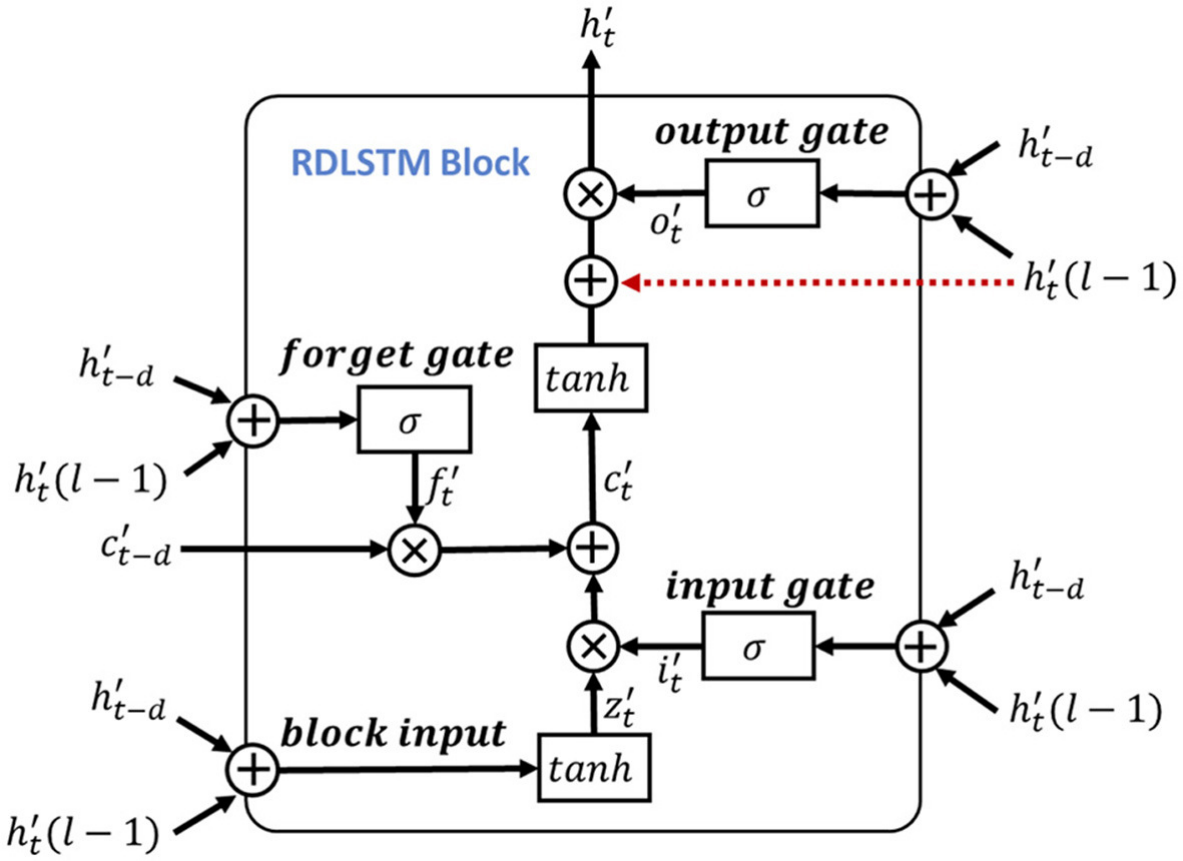
The structure of RDLSTM block.

To leverage the valuable information provided by the dependencies among multi-length features, the learned features obtained from the positional feature encoding are fed into the multi-length feature learning process. To be more specific, we denote the output features from the positional feature encoding process as **F_pos_**, for learning dependencies of the input signal features, we use the LSTM as the primary feature extraction unit for learning high-level representations, as illustrated in Figure 4. Notably, considering the shortcoming of LSTM of disappearing and losing the information of cell state (Schoene et al., 2020), we further add the residual dilation (Chang et al., 2017) to the LSTM for learning the multi-length and long sequence dependencies as shown in Figure 5. Here, denote the cell state, hidden state, and input of LSTM at time **t** as **c_t_**, **h_t_**, **x_t_**, respectively. Thus, the output of the block input **z**_*t*_ is calculated as:


(6) $$\begin{array}{l} {\text{z}}_{t}=g\left({\text{W}}_{z}{\text{x}}_{t}+{\text{R}}_{z}{\text{h}}_{t-1}+{\text{b}}_{z}\right) \end{array}$$

where **W**_*z*_, **R**_*z*_, **b**_*z*_ is the input weight, recurrent weight, and bias weight, respectively. The function of *g*(·) is the tanh activation function.

Then, the input gate, forget gate and output gate could be formulated as:


(7) $$\begin{array}{l} {\text{i}}_{t}=\sigma \left({\text{W}}_{i}{\text{x}}_{t}+{\text{R}}_{i}{\text{h}}_{t-1}+{\text{b}}_{i}\right) \end{array}$$

(8) $$\begin{array}{l} {\text{c}}_{t}={\text{f}}_{t}⊙{\text{c}}_{t-1}+{\text{i}}_{t}⊙{\text{z}}_{t} \end{array}$$

(9) $$\begin{array}{l} {\text{f}}_{t}=\sigma \left({\text{W}}_{f}{\text{x}}_{t}+{\text{R}}_{f}{\text{h}}_{t-1}+{\text{b}}_{f}\right) \end{array}$$

(10) $$\begin{array}{l} {\text{o}}_{t}=\sigma \left({\text{W}}_{o}{\text{x}}_{t}+{\text{R}}_{o}{\text{h}}_{t-1}+{\text{b}}_{o}\right) \end{array}$$

(11) $$\begin{array}{l} {\text{h}}_{t}=g\left({\text{c}}_{t}\right)⊙{\text{o}}_{t} \end{array}$$

here, the **W**_*i*_, **W**_*f*_, **W**_*o*_ and **b**_*i*_, **b**_*f*_, **b**_*o*_ are the corresponding inputs and bias weights of LSTM. The σ(·) and ⊙ represent the sigmoid and element-wise multiplication, respectively. In the RDLSTM, instead of using the previous cell state **c_t−1_** and hidden state **h_t−1_**, it takes in the cell state **c_t−d_** and hidden state **h_t−d_**, where the dilation rate **d** exponentially increases the receptive field of the LSTM. By incorporating these distant past states, the RDLSTM can capture long dependencies from the EEG signals, allowing for a more comprehensive understanding of the input sequence. Mathematically, we denote ${\text{z}}_{t}^{\prime }$, ${\text{c}}_{t}^{\prime }$, ${\text{h}}_{t}^{\prime }$, ${\text{i}}_{t}^{\prime }$, ${\text{f}}_{t}^{\prime }$, ${\text{o}}_{t}^{\prime }$ as the outputs of block input, cell state, hidden state, input gate, forget gate, and output gate, respectively. Then, the dilated LSTM could be defined as:


(12) $$\begin{array}{l} {\text{z}}_{t}^{\prime }=g\left({\text{W}}_{z}^{\prime }{\text{h}}_{t}^{\prime }\left(l-1\right)+{\text{R}}_{z}^{\prime }{\text{h}}_{t-d}+{\text{b}}_{z}^{\prime }\right) \end{array}$$

(13) $$\begin{array}{l} {\text{c}}_{t}^{\prime }={\text{f}}_{t}^{\prime }⊙{\text{c}}_{t-d}+{\text{i}}_{t}^{\prime }⊙{\text{z}}_{t}^{\prime } \end{array}$$

(14) $$\begin{array}{l} {\text{h}}_{t}^{\prime }={\text{o}}_{t}^{\prime }⊙\left(g\left({\text{c}}_{t}^{\prime }\right)+{\text{h}}_{t}^{\prime }\left(l-1\right)\right) \end{array}$$

(15) $$\begin{array}{l} {\text{i}}_{t}^{\prime }=\sigma \left({\text{W}}_{i}^{\prime }{\text{h}}_{t}^{\prime }\left(l-1\right)+{\text{R}}_{i}^{\prime }{\text{h}}_{t-d}^{\prime }+{\text{b}}_{i}^{\prime }\right) \end{array}$$

(16) $$\begin{array}{l} {\text{f}}_{t}^{\prime }=\sigma \left({\text{W}}_{f}^{\prime }{\text{h}}_{t}^{\prime }\left(l-1\right)+{\text{R}}_{f}^{\prime }{\text{h}}_{t-d}^{\prime }+{\text{b}}_{f}^{\prime }\right) \end{array}$$

(17) $$\begin{array}{l} {\text{o}}_{t}^{\prime }=\sigma \left({\text{W}}_{o}^{\prime }{\text{h}}_{t}^{\prime }\left(l-1\right)+{\text{R}}_{o}^{\prime }{\text{h}}_{t-d}^{\prime }+{\text{b}}_{o}^{\prime }\right) \end{array}$$

where ${\text{W}}_{z}^{\prime }$, ${\text{W}}_{i}^{\prime }$, ${\text{W}}_{f}^{\prime }$, ${\text{W}}_{o}^{\prime }$ are the input weights, ${\text{R}}_{z}^{\prime }$, ${\text{R}}_{i}^{\prime }$, ${\text{R}}_{f}^{\prime }$, ${\text{R}}_{o}^{\prime }$ denote the recurrent weights, and ${\text{b}}_{z}^{\prime }$, ${\text{b}}_{i}^{\prime }$, ${\text{b}}_{f}^{\prime }$, ${\text{b}}_{o}^{\prime }$ are the bias. The ${\text{h}}_{t}^{\prime }\left(l-1\right)$ represents the hidden state at the (*l* − 1)-th layer and is used to create a shortcut connection with the current LSTM cell to mitigate the issue of gradient vanishing. Additionally, hierarchically stacking the RDLSTMs enables the network to capture effective long dependencies among multi-length features across different layers. Therefore, various dilated rates of 1, 2, 4 are utilized to increase the receptive field of the network exponentially, enabling it to capture both local and global information. Following the processing in the RDLSTMs, the learned features are fed into the mutual-attention feature reinforcement process to extract global context information and further enhance the network's understanding of the input feature.

### 2.3 Mutual-attention feature reinforcement

**Figure 6:**
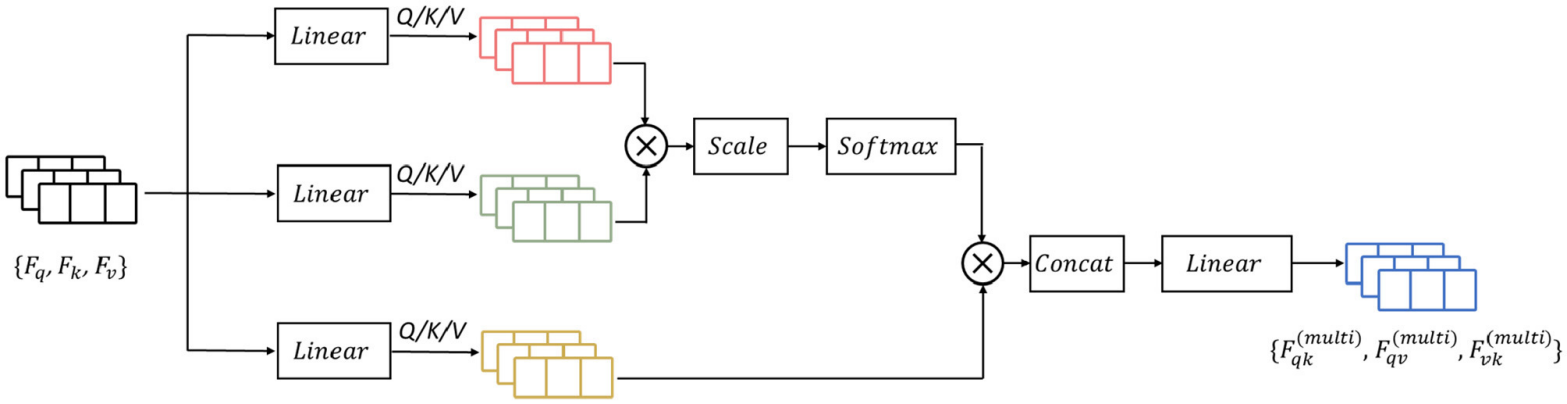
The structure of the mutual-attention feature reinforcement.

Previous research has demonstrated that attention-based learning is effective in encoding discriminative features and capturing global dependencies (Vaswani et al., 2017; Zhang et al., 2020). Building on these findings, we propose to enhance the feature learning capability by incorporating a mutual-attention feature reinforcement after the multi-length feature learning process (as shown in Figure 6). Formally, let the output feature maps of the RDLSTM with dilation rates **q**, **k**, **v** be denoted as **F**_*q*_, **F**_*k*_, **F**_*v*_ (**q** ≠ **k** ≠ **v**). In order to standardize the features to the same value domain, a batch normalization layer **N**(·). Ioffe and Szegedy (2015) is initially applied to **F**_*q*_, **F**_*k*_, **F**_*v*_, yielding ${\text{F}}_{q}^{\prime }$, ${\text{F}}_{k}^{\prime }$, ${\text{F}}_{v}^{\prime }$. Subsequently, these features with different value domains are separately passed through three linear layers and combined as inputs to the mutual-attention module. The formulation of this module can be expressed as:


(18) $$\begin{array}{l} \text{Q}={\text{W}}_{\text{q }}^{\left(query\right)}{\text{F}}_{q}^{\prime }+{\text{b}}_{\text{q }}^{\left(query\right)} \end{array}$$

(19) $$\begin{array}{l} \text{K}={\text{W}}_{\text{k }}^{\left(key\right)}{\text{F}}_{k}^{\prime }+{\text{b}}_{\text{k }}^{\left(key\right)} \end{array}$$

(20) $$\begin{array}{l} \text{V}={\text{W}}_{\text{v }}^{\left(value\right)}{\text{F}}_{v}^{\prime }+{\text{b}}_{\text{v }}^{\left(value\right)} \end{array}$$

where **Q**, **K**, **V** is the corresponding query, key, and value of ${\text{F}}_{q}^{\prime }$, ${\text{F}}_{k}^{\prime }$, ${\text{F}}_{v}^{\prime }$, ${\text{W}}_{\text{q}}^{\left(query\right)}$, ${\text{W}}_{\text{k}}^{\left(key\right)}$, and ${\text{W}}_{\text{v}}^{\left(value\right)}$ is the respective learned parameters of the linear operation, ${\text{b}}_{\text{q}}^{\left(query\right)}$, ${\text{b}}_{\text{k}}^{\left(key\right)}$, and ${\text{b}}_{\text{v}}^{\left(value\right)}$ is the bias, separately. Then, the mutual scaled-attention **A**_*qk*_,**A**_*qv*_,**A**_*vk*_ could be calculated by:


(21) $$\begin{array}{l} {\text{A}}_{qk}=\sigma \left(\frac{\text{Q}{\text{K}}^{⊺}}{\sqrt{S}}\right)\text{V} \end{array}$$

(22) $$\begin{array}{l} {\text{A}}_{qv}=\sigma \left(\frac{\text{Q}{\text{V}}^{⊺}}{\sqrt{S}}\right)\text{K} \end{array}$$

(23) $$\begin{array}{l} {\text{A}}_{vk}=\sigma \left(\frac{\text{V}{\text{K}}^{⊺}}{\sqrt{S}}\right)\text{Q} \end{array}$$

where $\sqrt{S}$ is the scaling parameter, ⊺ denotes the transpose, and the softmax function is performed on the gained attention values to normalize the attention values into probability distributions, which is defined as:


(24) $$\begin{array}{l} \sigma {\left(\text{z}\right)}_{d}=\frac{e^{{\text{z}}_{j}}}{\sum_{j=1}e^{{\text{z}}_{j}}} \end{array}$$

Furthermore, to exploit more discriminative and global representations, we employ the multi-head attention (Vaswani et al., 2017) which calculates the mutual-attention operations for *T* times. Thus, the output of multi-head attention ${\text{A}}_{d}^{\left(multi\right)}$ is formulated as:


(25) $$\begin{array}{l} {\text{A}}_{qk}^{\left(multi\right)}={\text{W}}_{qk}^{\left(\text{multi}\right)}\mathrm{Concat}\left({\text{A}}_{qk,1},{\text{A}}_{qk,2},\ldots ,{\text{A}}_{qk,T}\right) \end{array}$$

(26) $$\begin{array}{l} {\text{A}}_{qv}^{\left(multi\right)}={\text{W}}_{qv}^{\left(\text{multi}\right)}\mathrm{Concat}\left({\text{A}}_{qv,1},{\text{A}}_{qv,2},\ldots ,{\text{A}}_{qv,T}\right) \end{array}$$

(27) $$\begin{array}{l} {\text{A}}_{vk}^{\left(multi\right)}={\text{W}}_{vk}^{\left(\text{multi}\right)}\mathrm{Concat}\left({\text{A}}_{vk,1},{\text{A}}_{vk,2},\ldots ,{\text{A}}_{vk,T}\right) \end{array}$$

where ${\text{W}}_{qk}^{\left(\text{multi}\right)}$, ${\text{W}}_{qv}^{\left(\text{multi}\right)}$, ${\text{W}}_{vk}^{\left(\text{multi}\right)}$ are the weight matrices of the linear combination.

The operation of Concat(·) represents the concatenation of the input features. Finally, the output from mutual-attention feature reinforcement **F_at_** is given as:


(28) $$\begin{array}{l} {\text{F}}_{at}=Conv\left(Concat\left({\text{A}}_{qk}^{\left(multi\right)},{\text{A}}_{qv}^{\left(multi\right)},{\text{A}}_{vk}^{\left(multi\right)}\right)\right) \end{array}$$

### 2.4 Classification of EEG data

After the feature extraction process, the extracted features are passed into a softmax layer to generate prediction probabilities for different classes. Mathematically, the training data can be represented as {(**s**^(1)^, *y*^(1)^), (**s**^(2)^, *y*^(2)^), ⋯ , (**s**^(*N*)^, *y*^(*N*)^)}, where **s**^*N*^ ∈ ℝ^1×*C*^ denotes the input features, *y*^(*N*)^ ∈ {1, 2, ⋯ , *C*} represents the class label, and *C* is the total number of class labels. Therefore, the mapping function of softmax could be given as:


(29) $$\begin{array}{l} \begin{array}{c} h_{\theta }\left(\text{s}\right)=\left(\begin{array}{c} P\left(y=1|\text{s};\theta \right)\\ P\left(y=2|\text{s};\theta \right)\\ \vdots \\ P\left(y=C|\text{s};\theta \right) \end{array}\right)\\ =\frac{1}{\overset{K}{\underset{j=1}{\sum }}\exp\left(\theta_{j}^{T}\text{s}\right)}\left(\begin{array}{c} \exp\left(\theta_{1}^{T}\text{s}\right)\\ \exp\left(\theta_{2}^{T}\text{s}\right)\\ \vdots \\ \exp\left(\theta_{C}^{T}\text{s}\right) \end{array}\right) \end{array} \end{array}$$

where θ is the learned parameters of the softmax. To optimize the network, we use the cross-entropy ***J***(·) as the loss function, which can be defined as:


(30) $$\begin{array}{l} J\left(\text{w}\right)=-\frac{1}{N}\overset{C}{\underset{c=1}{\sum }}\left[{\text{y}}_{c}\log{\hat{y}}_{c}+\left(1-{\hat{y}}_{c}\right)\log\left(1-{\hat{y}}_{c}\right)\right] \end{array}$$

where **y_c_** is the true class label, and ${\hat{y}}_{c}$ denotes the predicted class label. Overall, the whole process of the proposed PMM Network could be illustrated in Algorithm 1.

**Algorithm 1:**
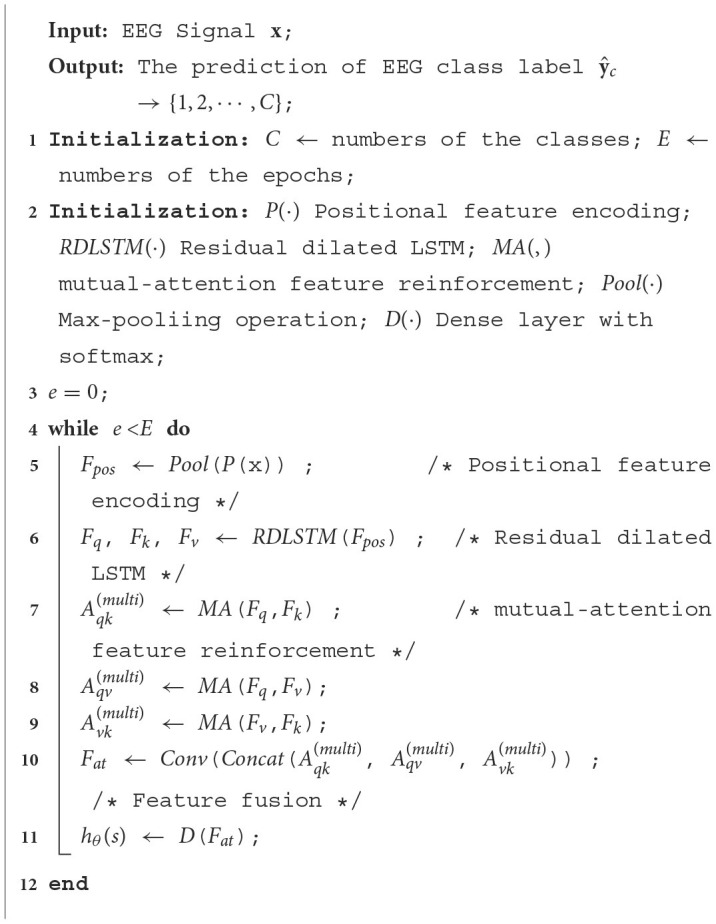
Epileptic Seizure Classification of PMM Network.

## 3 Data description

We evaluate our approach using the Bonn EEG dataset, initially reported in Andrzejak (2001). This dataset consists of five subsets: Set A, B, C, D, and E. Each subset contains 100 EEG channels and has a duration of 23.6 seconds. Subsets A and B were collected from healthy subjects, with recordings taken during both eyes open and closed conditions. Subsets C, D, and E correspond to different locations in epileptic subjects. Subset C represents recordings from the hippocampal formation, Subset D records the epileptogenic zone, and Subset E captures signals during seizure activity. It is important to note that the signals in subset C and D were recorded during seizure-free intervals, while subset E was captured during seizure activity. For simplicity, the eye movements in dataset A and B were not considered in our evaluation.

Moreover, the UCI-EEG Recognition dataset (Wu and Fokoue, 2017) is also used for the detection of epileptic seizures. It consists of five distinct groups, each consisting of 100 single-channel EEG signals. Each EEG file corresponds to a 23.6-s recording of brain activity, which is sampled into 4097 data points. Therefore, the dataset comprises a total of 500 subjects, with each subject's data containing 4,097 data points. Additionally, the EEG samples in this dataset are further divided into 23 data chunks, with each chunk containing 178 data points. Overall, the dataset contains 11,500 time-series EEG signal data samples from the 500 subjects. In the EEG recognition dataset, Class 1 represents the state of epileptic seizure, while Classes 2–5 represent normal healthy states. This dataset facilitates a binary classification task aimed at distinguishing between the combined normal states (Classes 2–5) and the seizure condition (Class 1).

## 4 Results

### 4.1 Implementation details

The PMM network is implemented using the PyTorch deep learning framework, and cross-entropy is adopted as the loss function. The optimizer used is Adam, which helps in the convergence of the network. The initial learning rate is set at 0.0003 and is decayed by a factor of 0.001 after each epoch. To accelerate the training process, a GTX 1080 GPU is used. Additionally, 10-fold cross-validation is carried out to assess the performance of the model.

### 4.2 Evaluation metrics

The performance of the experiment is evaluated using several commonly used performance metrics, including accuracy, precision, sensitivity, specificity, and F1-score.

Accuracy is defined as the ratio of the number of correctly predicted samples to the total number of predicted samples, which is defined as


(31) $$\begin{array}{l} Accuracy=\frac{TP+TN}{TP+TN+FP+FN} \end{array}$$

Precision refers to the ratio of the number of correctly predicted positive samples to the total number of predicted positive samples, which is given as


(32) $$\begin{array}{l} Precision=\frac{TP}{TP+FP} \end{array}$$

Sensitivity measures the proportion of positives that are correctly identified, which is defined as


(33) $$\begin{array}{l} Sensitivity=\frac{TP}{TP+FN} \end{array}$$

Specificity measures the proportion of negatives that are correctly identified, which is defined as


(34) $$\begin{array}{l} Specificity=\frac{TN}{FP+TN} \end{array}$$

F1 score is the harmonic mean of precision and sensitivity, which is defined as


(35) $$\begin{array}{l} F1-score=2\times \frac{Precision\times Sensitivity}{Precision+Sensitivity} \end{array}$$

Among all the equations presented above, the term TP (true positives) represents the number of EEG data samples that are abnormal and correctly identified as abnormal. Similarly, TN (true negatives) represents the number of EEG data samples that are normal and correctly identified as normal. FP (false positives) refers to the number of normal EEG data samples that are incorrectly predicted as abnormal, and FN (false negatives) refers to the number of abnormal EEG data samples that are incorrectly predicted as normal.

To ensure a comprehensive evaluation of the system, a 10-fold cross-validation approach is applied. During each iteration, one fold is used for testing the model, while the remaining nine folds are used for training. This process is repeated ten times, with each fold used as the test set once. The average values of accuracy, sensitivity, and specificity are then collected from the ten-fold cross-validation, providing an average performance measurement of the system across different categories of data.

### 4.3 The performance of double classes classification

In this section, the performance of the double class classification is evaluated on Bonn dataset. Table 1 compares the performance of different combinations of double classes, including A-E, B-E, C-E, D-E, AB-E, AC-E, AD-E, BC-E, BD-E, CD-E, ABC-E, ABD-E, BCD-E, and ABCD-E on Bonn dataset. Among these combinations, the highest performance is achieved in the A-E class classification with an accuracy of 99.95%, while the most challenging classification task is D-E with an accuracy of 98.79%. The experimental results reveal a significant difference between the eyes open classes and the seizure epileptic classes, resulting in a higher classification performance. Furthermore, these results demonstrate that our proposed PMM network performs well on different double class classification tasks.

**Table 1:** The overall performance of double classes classification on Bonn dataset.

	**Accuracy(%)**	**Sensitivity(%)**	**Specificity(%)**	**Precision(%)**	**F1-score(%)**
A-E	99.95	98.97	99.98	99.97	99.65
B-E	99.76	98.02	99.93	99.92	98.99
C-E	99.43	96.73	99.87	99.85	98.53
D-E	98.79	97.16	98.35	98.52	97.73
AB-E	99.82	97.90	99.99	99.97	98.97
AC-E	99.67	96.98	99.92	99.68	98.32
AD-E	99.23	97.34	99.52	98.96	97.78
BC-E	99.52	96.73	99.90	99.81	98.26
BD-E	98.85	95.76	99.53	98.96	97.52
CD-E	98.87	95.32	99.65	99.36	97.38
ABC-E	99.62	96.23	99.98	99.93	97.99
ABD-E	99.41	96.53	99.60	98.75	97.66
BCD-E	98.99	94.51	99.65	98.82	96.56
ABCD-E	99.23	94.72	99.81	99.01	96.86

### 4.4 The performance of multiple classes classification

We further evaluate the performance of the proposed PMM network on multiple class classification using Bonn dataset. We compare the combinations of classes including A-C-E, A-D-E, B-C-E, B-D-E, AB-CD-E, and A-B-C-D-E separately, and present the results in Table 2. The results clearly indicate that the B-D-E combination achieves the best performance, with an accuracy of 98.73%, sensitivity of 97.22%, specificity of 98.61%, precision of 97.32%, and F1-score of 97.52%. On the other hand, the A-B-C-D-E combination, consisting of five classes, shows the lowest performance. This showcases the increasing difficulty of multiple class classification tasks as the number of classes increases.

**Table 2:** The overall performance of multiple classes classification on Bonn dataset.

	**Accuracy(%)**	**Sensitivity(%)**	**Specificity(%)**	**Precision(%)**	**F1-score(%)**
A-C-E	97.63	95.61	97.82	95.53	95.51
A-D-E	97.83	95.90	97.96	95.90	95.88
B-C-E	98.53	97.02	98.62	97.12	97.02
B-D-E	98.73	97.22	98.61	97.32	97.52
AB-CD-E	97.78	95.98	97.93	95.92	95.91
A-B-C-D-E	94.71	83.51	83.33	95.86	89.61

### 4.5 The effectiveness of different components

In this section, we conduct extensive experiments to validate the effectiveness of each proposed component in the A-B-C-D-E classes combination classification task using Bonn dataset. We refer to the positional feature encoding block, multi-length feature learning, mutual-attention feature reinforcement, and residual dilation with LSTM as PFEBlock, MFL, MFR, and RDLSTM, respectively. The model without any proposed module is defined as “Original”. Table 3 illustrates the results of these experiments. It can be observed that integrating any of the processes, i.e., PFEBlock, MFL, or MFR, leads to improved classification performance compared to RDLSTM. This confirms the effectiveness of each proposed process in enhancing the overall classification performance. Additionally, we find that adding the MFL could gain the best performance, which further demonstrate that the dependencies among multi-length features play vital importance in this task.

**Table 3:** The effectiveness of different components.

	**Accuracy(%)**	**Sensitivity(%)**	**Specificity(%)**	**Precision(%)**	**F1-score(%)**
Original	92.08	81.52	80.32	93.02	86.55
+ RDLSTM	93.17	82.01	81.02	93.67	87.16
+ PFEBlock	93.68	82.23	81.16	94.01	87.88
+ MFL	94.19	83.02	82.56	95.02	89.01
+ MFR	93.92	82.63	81.78	94.26	88.30
Ours	94.71	83.51	83.33	95.86	89.61

### 4.6 Influence of different dilated rates

The optimal dilated rates in the Dilated LSTM network play a crucial role in achieving improved performance. Through our experiments, we conducte tests to determine the best rates based on various metrics. The results, as shown in Table 4, indicate that larger dilated rates lead to enhanced performance when using a single dilated rate or two different rates. The higher dilated rates offer better performance because they allow the network to capture a broader range of information from the input data. By increasing the dilation rate, the network can expand its receptive field and consider a wider context, resulting in more accurate and informed predictions. Comparing the dilated rate sequences “1, 2, 4, 8” and “1, 2, 4”, we find that the latter sequence achieves superior performance. This is because the rates “1, 2, 4” strike a balance between capturing local patterns and incorporating global relationships within the data. On the other hand, including the rate “8” in the first sequence potentially introduces noise or redundant information, which may degrade the model's performance.

**Table 4:** The influence of different dilated rates on Bonn dataset.

	**Accuracy(%)**	**Sensitivity(%)**	**Specificity(%)**	**Precision(%)**	**F1-score(%)**
d = 1	93.21	81.65	80.45	93.22	86.78
d = 2	93.73	82.01	80.67	93.53	87.03
d = 4	93.97	82.26	81.12	93.88	87.32
d = 1,2	94.14	82.78	81.68	94.25	87.79
d = 1,4	94.41	83.13	82.23	94.62	88.25
d = 2,4	94.56	83.49	83.21	95.10	89.13
d = 1,2,4	94.71	83.51	83.33	95.86	89.61
d = 1,2,4,8	94.65	83.45	83.31	95.78	89.58

### 4.7 Compare with other classification methods

To evaluate the performance of the proposed network, we first compare it with other classification methods on Bonn dataset, especially those based on CNN, in various classification tasks. For consistency, we use fixed combinations of EEG classes, including ABCD-E, AB-CD-E, A-B-C-D-E, A-E, AC-E, C-E, A-D-E, D-E, A-D, B-E, and B-C-D. Tables 5, 6 present the results of the comparison. It can be observed that our proposed method achieves competitive performance in most classification tasks when compared to other methods, particularly in the double classes classification task. This demonstrates the effectiveness of our proposed method in handling and accurately classifying EEG signals for various classification tasks. In addition, we conduct experiments on binary classification using the UCI-EEG dataset, and the experimental results are illustrated in Table 7. The results demonstrate that our proposed method achieved the highest classification accuracy compared to other classification methods.

**Table 5:** Comparison with other methods (a).

**Category**	**Method**	**Accuracy(%)**
ABCD-E	KST&MWUT+AB-BP-NN Al-Hadeethi et al. (2021)	98.5
	GA-SVM Zhang and Chen (2016)	98.9
	Ours	99.2
AB-CD-E	DWT+SVM/KNN/NB/DT Kavitha et al. (2022)	95.0
	GA-SVM Zhang and Chen (2016)	98.4
	XGBoost Liu (2023)	89.0
	Ours	97.8
A-B-C-D-E	LSP-SVM Tuncer et al. (2019)	93.0
	CWT + CNN Türk and Özerdem (2019)	93.6
	CE-stSENet Li et al. (2020)	94.6
	ResNet-LSTM Qiu et al. (2023)	90.2
	Ours	94.7
A-E	CWT + CNN Türk and Özerdem (2019)	99.5
	HT+LS-SVM Siuly et al. (2019)	99.5
	ANFIS-BS Shoeibi et al. (2022)	99.8
	2D CNN STFT + LSTM Varli and Yilmaz (2023)	99.8
	WNFG-GNN-ADMN Wang et al. (2023)	100.0
	GA-SVM Zhang and Chen (2016)	100.0
	Ours	100.0
AC-E	DWT+SVM/KNN/NB/DT Kavitha et al. (2022)	98.7
	HT+LS-SVM Siuly et al. (2019)	99.7
	Ours	99.7
C-E	DWT+SVM/KNN/NB/DT Kavitha et al. (2022)	98.4
	CWT + CNN Türk and Özerdem (2019)	98.5
	HT+LS-SVM Siuly et al. (2019)	98.5
	KST&MWUT+AB-BP-NN Al-Hadeethi et al. (2021)	98.5
	WNFG-GNN-ADMN Wang et al. (2023)	98.4
	Ours	99.4
A-D-E	PSD-SVM Riaz (2015)	85.0
	WPD + ETTL-TSK-FS DengZ (2018)	95.7
	Ours	97.8
A-D	WPD + ETTL-TSK-FS DengZ (2018)	97.5
	LSP-SVM Tuncer et al. (2019)	99.5
	Ours	99.7

**Table 6:** Comparison with other methods (b).

**Category**	**Method**	**Accuracy(%)**
D-E	HT+LS-SVM Siuly et al. (2019)	97.5
	GA-SVM Zhang and Chen (2016)	98.1
	VMD + SampEn Das et al. (2018)	98.8
	WNFG-GNN-ADMN Wang et al. (2023)	97.2
	2D CNN STFT + LSTM Varli and Yilmaz (2023)	98.1
	Ours	98.8
AB-E	HT+LS-SVM Siuly et al. (2019)	98.67
	VMD + SampEn Das et al. (2018)	100.0
	Ours	100.0
B-E	LSP-SVM Tuncer et al. (2019)	96.5
	WPD + ETTL-TSK-FS DengZ (2018)	97.0
	HT+LS-SVM Siuly et al. (2019)	99.0
	ANFIS-BS Shoeibi et al. (2022)	99.8
	WNFG-GNN-ADMN Wang et al. (2023)	99.7
	Ours	99.8
B-C-D	WPD + ETTL-TSK-FS DengZ (2018)	95.0
	Ours	96.1

**Table 7:** Comparison with other methods on UCI-EEG dataset.

**Category**	**Method**	**Accuracy(%)**
Normal-seizure	SMARTSeiz Patro et al. (2023)	99.6
	Attention-CNN Ahmad et al. (2024)	99.6
	MGRU Prakash and Kumar (2023)	98.8
	ANN Rohan et al. (2020)	98.3
	Ours	99.8

## 5 Discussion

From a technical perspective, our paper introduces the PMM network as a potentional solution to address the challenges associated with learning minute abnormal characteristics and modeling long dependencies in EEG signals. We employ positional feature encoding to enhance the network's detection of subtle abnormalities, leveraging temporal position information. Additionally, our proposed multi-length feature learning enables the network to extract features at different scales, capturing short-term and long-term dependencies in the EEG signals. Moreover, incorporating the mutual-attention feature reinforcement mechanism enhances the network's ability to identify relevant spatial and temporal dependencies, allowing it to distinguish abnormal patterns from background activity more effectively. These advancements collectively contribute to the PMM network's potential in clinical applications and EEG signal analysis by providing a more comprehensive and accurate approach for capturing small abnormal characteristics, modeling long dependencies, and improving attention mechanisms.

From a clinical perspective, our proposed PMM network offers improved advancements in EEG signal analysis that have the potential to benefit clinical practice. It effectively captures minute abnormal characteristics often associated with neurological disorders, allowing for precise identification even within complex EEG patterns. By modeling long dependencies and incorporating multi-length feature learning, the network provides a comprehensive understanding of the underlying abnormal processes that evolve over time. The mutual-attention feature reinforcement mechanism further enhances specificity in detecting abnormal patterns, which is crucial for accurate diagnosis and informed decision-making in patient management. While these improvements hold promise for clinical practice, it is important to note that further research and evaluation are needed. Extensive testing with diverse datasets is necessary to validate the network's performance across various EEG classification tasks encountered in real-world clinical settings. Such validation is crucial before the network's potential can be fully realized and integrated into routine clinical workflows.

## 6 Conclusion

In this paper, we introduced the PMM network to address the challenges related to learning minute abnormal characteristics and long dependencies in EEG signals. Our proposed approach effectively captures the minute abnormal characteristics through positional feature encoding and improves the modeling of long dependencies with multi-length feature learning and mutual-attention feature reinforcement. Experimental evaluations on the publicly available dataset demonstrated that the PMM network achieves competitive performance compared to other state-of-the-art methods. One limitation of this study is that the proposed network was only evaluated on the limited dataset, which may not cover the full spectrum of EEG classification tasks. Therefore, in future work, we aim to extend our network to more diverse time-series datasets to further validate its effectiveness and generalizability. Moreover, while this approach proves beneficial for capturing subtle abnormal characteristics in EEG signals, it may be sensitive to variations in signal alignment and time-dependent patterns. Different EEG recording setups or variations in patient-specific factors could introduce spatial and temporal misalignments, potentially affecting the network's performance. Therefore, future research should focus on developing more robust techniques for positional feature encoding that can adapt to different recording setups and account for these variations, ensuring the network's stability and reliability across diverse EEG datasets. By addressing this limitation, we can enhance the network's applicability and strengthen its performance in real-world clinical scenarios.

## Data availability statement

Publicly available datasets were analyzed in this study. This data can be found here: https://www.upf.edu/web/ntsa/downloads.

## Author contributions

GZ: Writing—original draft, Writing—review & editing. AZ: Writing—review & editing. HL: Writing—review & editing. JL: Writing—review & editing. JC: Writing—review & editing.

## References

[B1] Ahmad I. Yao C. Li L.. (2024). An efficient feature selection and explainable classification method for EEG-based epileptic seizure detection. J. Inform. Commun. Technol. 80, 103654. 10.1016/j.jisa.2023.103654

[B2] Al-Hadeethi H. Abdulla S. Diykh M. Green J. H. (2021). Determinant of covariance matrix model coupled with adaboost classification algorithm for EEG seizure detection. Diagnostics 12, 74. 10.3390/diagnostics12010074 35054242 PMC8774996

[B3] Aliyu I. Lim C. G. (2021). Selection of optimal wavelet features for epileptic EEG signal classification with LSTM. Neural Comp. Appl. 2021, 1–21. 10.1007/s00521-020-05666-0

[B4] Andrzejak R. G. Lehnertz K. Mormann F. Rieke C. David P. Elger C. E. (2001). Indications of nonlinear deterministic and finite-dimensional structures in time series of brain electrical activity: dependence on recording region and brain state. Phys. Rev. E 64, 061907. 10.1103/PhysRevE.64.061907 11736210

[B5] Chai X. Wang Q. Zhao Y. Liu X. Bai O. Li Y. (2016). Unsupervised domain adaptation techniques based on auto-encoder for non-stationary EEG-based emotion recognition. Comp. Biol. Med. 79, 205–214. 10.1016/j.compbiomed.2016.10.019 27810626

[B6] Chang S. Zhang Y. Han W. Yu M. Guo X. Tan W.. (2017). Dilated recurrent neural networks. Adv. Neural Inf. Process Syst. 30, 77–87.

[B7] Chen J. Guo Z. Xu X. Zhang L. Teng Y. Chen Y.. (2023). Robust deep learning framework based on spectrograms for heart sound classification. IEEE/ACM Trans Comput Biol Bioinform. 22, 433. 10.1109/TCBB.2023.3247433 37027654

[B8] Chen J. Sun S. Zhang L. Yang B. Wang W. (2021). Compressed sensing framework for heart sound acquisition in internet of medical things. IEEE Trans. Indust. Informat. 18, 2000–2009. 10.1109/TII.2021.3088465

[B9] Das P. Manikandan M. S. Ramkumar B. (2018). Detection of epileptic seizure event in EEG signals using variational mode decomposition and mode spectral entropy, in 2018 IEEE 13th International Conference on Industrial and Information Systems (ICIIS). Rupnagar: IEEE, 42–47. 10.1109/ICIINFS.2018.8721426

[B10] Deng Z. Xu P. Xie L. Choi K.-S. Wang S. (2018). Transductive joint-knowledge-transfer TSK FS for recognition of epileptic EEG signals. IEEE Trans. Neural Syst.d Rehabilit. Eng. 26, 1481–1494. 10.1109/TNSRE.2018.2850308 29994680

[B11] Gao Z. Cui X. Wan W.. (2019). Recognition of emotional states using multiscale information analysis of high frequency EEG oscillations. Entropy 21, 609. 10.3390/e21060609 33267323 PMC7515095

[B12] Hossain M. S. Amin S. U. Alsulaiman M. Muhammad G. (2019). Applying deep learning for epilepsy seizure detection and brain mapping visualization. ACM Trans. Multimedia Comp. Commun. Applicat. (TOMM). 15, 1–17. 10.1145/3241056

[B13] Hussain W. Sadiq M. T. Siuly S. Rehman A. (2021). Epileptic seizure detection using 1 D-convolutional long short-term memory neural networks. Appl. Acoust. 177, 107941. 10.1016/j.apacoust.2021.107941

[B14] Ioffe S. Szegedy C. (2015). Batch normalization: Accelerating deep network training by reducing internal covariate shift, in International Conference on Machine Learning (New York, NY: PMLR), 448–456.

[B15] Jrad N. Kachenoura A. Merlet I. Bartolomei F. Nica A. Biraben A. (2016). Automatic detection and classification of high-frequency oscillations in depth-EEG signals. IEEE Trans. Biomed. Eng, 64, 2230–2240. 10.1109/TBME.2016.2633391 28113293

[B16] Kasabov N. Capecci E. (2015). Spiking neural network methodology for modelling, classification and understanding of EEG spatio-temporal data measuring cognitive processes. Inform. Sci. 294, 565–575. 10.1016/j.ins.2014.06.028

[B17] Kavitha K. V. N. Ashok S. Imoize A. L. Ojo S. Selvan K. Ahanger T.. (2022). On the use of wavelet domain and machine learning for the analysis of epileptic seizure detection from EEG signals. J. Healthc Eng. 2022, 8928021. 10.1155/2022/8928021 35251581 PMC8896918

[B18] Lemm S. Blankertz B. Curio G. Muller K. (2005). Spatio-spectral filters for improving the classification of single trial EEG. IEEE Trans. Biomed. Eng. 52, 1541–1548. 10.1109/TBME.2005.851521 16189967

[B19] Li Y. Liu Y. Cui W.-G. Guo Y.-Z. Huang H. Hu Z.-Y. (2020). Epileptic seizure detection in EEG signals using a unified temporal-spectral squeeze-and-excitation network. IEEE Trans. Neural Syst. Rehabilitat. Eng. 28, 782–794. 10.1109/TNSRE.2020.2973434 32078551

[B20] Liu J. Wu G. Luo Y. Qiu S. Yang S. Li W.. (2020). EEG-based Emotion Classification Using Deep Neural Network and Sparse Autoencoder. Front. Syst. Neurosci. 14, 43. 10.3389/fnsys.2020.00043 32982703 PMC7492909

[B21] Liu M. Zhou M. Zhang T.. (2020). Semi-supervised learning quantization algorithm with deep features for motor imagery EEG Recognition in smart healthcare application. Appl. Soft Comp. 89, 106071. 10.1016/j.asoc.2020.106071

[B22] Liu Q. Wang Z. Dong H. Jiang C. (2022). Remote estimation for energy harvesting systems under multiplicative noises: a binary encoding scheme with probabilistic bit flips. IEEE Trans. Automatic Control. 68, 3170540. 10.1109/TAC.2022.3170540

[B23] Liu Z. Zhu B. Hu M. Deng Z. Zhang J. (2023). Revised tunable q-factor wavelet transform for EEG-based epileptic seizure detection. IEEE Trans. Neural Syst. Rehabilit. Eng. 31, 1707–1720. 10.1109/TNSRE.2023.3257306 37028382

[B24] Meng J. Yao L. Sheng X. Zhang D. Zhu X. (2014). Simultaneously optimizing spatial spectral features based on mutual information for EEG classification. Trans. Biomed. Eng. 62, 227–240. 10.1109/TBME.2014.2345458 25122834

[B25] Patro K. K. Prakash A. J. Sahoo J. P. Routray S. Baihan A. Samee N.. (2023). SMARTSeiz: deep learning with attention mechanism for accurate seizure recognition in iot healthcare devices. IEEE J Biomed Health Inform. 6, 3336935. 10.1109/JBHI.2023.3336935 38055360

[B26] Prakash V. Kumar D. A. (2023). Modified gated recurrent unit approach for epileptic electroencephalography classification. J. Inform. Commun. Technol. 22, 587–617. 10.32890/jict2023.22.4.3

[B27] Qi F. Li Y. Wu W. R. (2015). A novel algorithm for spatio-temporal filtering and classification of single-trial EEG. IEEE Trans. Neural Networks Learning Syst. 26, 3070–3082. 10.1109/TNNLS.2015.2402694 25730834

[B28] Qiu X. Yan F. Liu H. A. (2023). difference attention ResNet-LSTM network for epileptic seizure detection using EEG signal. Biomed. Signal Proc. Control 83, 104652. 10.1016/j.bspc.2023.104652

[B29] Qiu Y. Zhou W. Yu N. Du P. (2018). Denoising sparse autoencoder-based ictal EEG classification. IEEE Trans. Neural Syst. Rehabilit. Eng. 26, 1717–1726. 10.1109/TNSRE.2018.2864306 30106681

[B30] Rajinikanth V. Kadry S. Taniar D. Kamaland K. Elaziz M. A. Palani K. (2022). Detecting epilepsy in EEG signals using synchro-extracting-transform (SET) supported classification technique. J. Ambient Intellig. Human. Comp. 2022, 1–19. 10.1007/s12652-021-03676-x

[B31] Riaz F. Hassan A. Rehman S. Niazi I. K. Dremstrup K. (2015). EMD-based temporal and spectral features for the classification of EEG signals using supervised learning. IEEE Trans. Neural Syst. Rehabilitat. Eng 24, 28–35. 10.1109/TNSRE.2015.2441835 26068546

[B32] Rohan T. I. Yusuf M. S. U. Islam M. Roy S. (2020). Efficient approach to detect epileptic seizure using machine learning models for modern healthcare system. IEEE. 2020, 1783–1786. 10.1109/TENSYMP50017.2020.9230731

[B33] Saichand N. V. (2021). Epileptic seizure detection using novel multilayer LSTM discriminant network and dynamic mode Koopman decomposition. Biomed. Signal Proc. Control 68, 102723. 10.1016/j.bspc.2021.102723

[B34] Schoene A. M. Turner A. Dethlefs N. (2020). Bidirectional dilated LSTM with attention for fine-grained emotion classification in tweets, in Proceedings of the AAAI-20 Workshop on Affective Content Analysis (New York, USA: AAAI).

[B35] Shoeibi A. Ghassemi N. Khodatars M.. (2022). Detection of epileptic seizures on EEG signals using ANFIS classifier, autoencoders and fuzzy entropies. Biomed. Signal Proc. Control 73, 103417. 10.1016/j.bspc.2021.103417

[B36] Siuly S. Alcin O. F. Bajaj V.. (2019). Exploring Hermite transformation in brain signal analysis for the detection of epileptic seizure. IET Sci. Measur. Technol.13, 35–41. 10.1049/iet-smt.2018.5358

[B37] Tsipouras M. G. (2019). Spectral information of EEG signals with respect to epilepsy classification. EURASIP 2019, 1–17. 10.1186/s13634-019-0606-8

[B38] Tuncer T. Dogan S. Akbal E. A. (2019). novel local senary pattern based epilepsy diagnosis system using EEG signals. Aust. Phys. Eng. Sci. Med. 42, 939–948. 10.1007/s13246-019-00794-x 31482442

[B39] Türk Ö. Özerdem M. S. (2019). Epilepsy detection by using scalogram based convolutional neural network from EEG signals. Brain Sci. 9, 115. 10.3390/brainsci9050115 31109020 PMC6562774

[B40] Varli M. Yilmaz H. (2023). Multiple classification of EEG signals and epileptic seizure diagnosis with combined deep learning. J. Comp. Sci. 67, 101943. 10.1016/j.jocs.2023.101943

[B41] Vaswani A. Shazeer N. Parmar N. Uszkoreit J. Jones L. Gomez A. N.. (2017). Attention is all you need, in Advances in Neural Information Processing Systems, 5998–6008.

[B42] Wang J. Gao R. Zheng H. Zhu H. Shi C-J. R. (2023). SSGCNet: a sparse spectra graph convolutional network for epileptic EEG signal classification. IEEE Trans. Neural. Netw. Learn. Syst. 16, 3252569. 10.1109/TNNLS.2023.3252569 37030729

[B43] Wu D. Shi Y. Wang Z. Yang J. Sawan M. (2023). C 2 SP-Net: joint compression and classification network for epilepsy seizure prediction. IEEE Trans. Neural Syst. Rehabilitat. Eng. 31, 841–850. 10.1109/TNSRE.2023.3235390 37018579

[B44] Wu Q. Fokoue E. (2017). Epileptic seizure recognition, in UCI Machine Learning Repository.

[B45] Xin Q. Hu S. Liu S. Zhao L. Zhang Y.-D. (2022). An attention-based wavelet convolution neural network for epilepsy EEG classification. IEEE Trans. Neural Syst. Rehabilit. Eng. 30, 957–966. 10.1109/TNSRE.2022.3166181 35404819

[B46] Yuan Y. Xun G. Jia K. Zhang A. (2018a). A multi-view deep learning framework for EEG seizure detection. IEEE J. Biomed. Health Informat. 23, 83–94. 10.1109/JBHI.2018.2871678 30624207

[B47] Yuan Y. Xun G. Ma F. Suo Q. Xue H. Jia K.. (2018b). A novel channel-aware attention framework for multi-channel eeg seizure detection via multi-view deep learning, in 2018 IEEE EMBS International Conference on Biomedical and Health Informatics (BHI) (Las Vegas, NV: IEEE), 206–209. 10.1109/BHI.2018.8333405

[B48] Zhang G. Le Yang B. Li B. Lu Y. Liu Q. Zhao W.. (2020). MNL-network: a multi-scale non-local network for epilepsy detection from EEG signals. Front. Neurosci. 14, 870. 10.3389/fnins.2020.00870 33281538 PMC7705239

[B49] Zhang T. Chen W. L. M. D. (2016). based features for the automatic seizure detection of EEG signals using SVM. IEEE Trans. Neural Syst. Rehabilitat. Eng. 25, 1100–1108. 10.1109/TNSRE.2016.2611601 27662677

[B50] Zheng W. L. Lu B. L. (2015). Investigating critical frequency bands and channels for EEG-based emotion recognition with deep neural networks. IEEE Trans. Auton. Mental Dev. 7, 162–175. 10.1109/TAMD.2015.2431497

